# Association between immune cells and urticaria: a bidirectional Mendelian randomization study

**DOI:** 10.3389/fimmu.2024.1439331

**Published:** 2024-11-13

**Authors:** Yongjun Chen, Xuejie Chen, Zhipeng Zhang

**Affiliations:** ^1^ Department of Dermatology, Huangshi Central Hospital, Affiliated Hospital of Hubei Polytechnic University, Huangshi, China; ^2^ Department of Plastic Surgery, Renmin Hospital of Wuhan University, Wuhan, Hubei, China; ^3^ Xianning Medical College, Hubei University of Science & Technology, Xianning, Hubei, China

**Keywords:** urticaria, immune cells, bidirectional Mendelian randomization, instrumental variables, lymphocytes

## Abstract

Urticaria is characterized by transient itchy symptoms on the skin, usually accompanied by swelling, which is caused by mast cell activation leading to increased vascular permeability and dilation of the dermis. Urticaria involves recurrent activation of mast cells, T cells, eosinophils, and other immune cells around lesioned venules, with complex regulatory systems affecting mast cell functions, potentially contributing to urticaria pathogenesis. The direct causal relationship between immune cells and urticaria is currently unclear. To address this, our study utilized a bidirectional Mendelian randomization analysis, employing instrumental variables (IVs) associated with immune cells and urticaria, to investigate this causal relationship. First, by utilizing Genome-wide Association Study (GWAS) data, we identified 31 immunophenotypes associated with urticaria risk, with 18 increasing and 13 decreasing the risk. Through rigorous criteria, we identified 4 immunophenotypes that have a strong causal relationship with urticaria. Notably, HLA DR+ CD4+AC, CD45 on CD8br, and HLA DR on plasmacytoid dendritic cells were associated with an increased risk, while CD8dim NKT %lymphocyte was identified as a protective factor. Sensitivity analyses, including the MR-Egger intercept test, scatter plots, funnel plots, and leave-one-out analysis, supported the robustness of the findings. Reverse MR analysis suggested an inverse causal effect of urticaria on CD8dim NKT %lymphocyte, reinforcing the potential bidirectional nature of the relationship between urticaria and immune cell phenotypes. Our research substantiates the bidirectional causal relationship between immune cells and urticaria, thus benefiting for urticaria-targeted therapy development.

## 1 Introduction

Urticaria, commonly referred to as hives, manifests as a transient, itchy skin condition characterized by raised, red welts that result from vascular dilation and enhanced capillary permeability, primarily mediated by mast cell activation (1, 2). Urticaria can significantly impact the quality of life, causing discomfort and distress due to persistent itching and the unpredictability of flare-ups (3, 4). Urticaria can be triggered by factors including certain drugs and foods, physical stimuli, infections, stress, endocrine changes, and various internal diseases (5, 6). Numerous studies have demonstrated that the risk of developing urticaria has a substantial genetic component, suggesting that hereditary factors play a significant role in the susceptibility to this condition (7). Continued exploration into the pathogenesis of urticaria will contribute to the development of more effective treatment strategies (8).

The immune system are crucial participants and shapers in the etiology and progression of diseases, particularly in dermatological disorders (9, 10). Urticaria is associated with immune responses, particularly Type I hypersensitivity reactions, where immunoglobulin E (IgE) plays a crucial role in triggering inflammation, leading to vascular dilation, increased permeability, smooth muscle contraction, and enhanced glandular secretion (4, 11, 12). The development and manifestation of urticaria are deeply rooted in immunopathological processes, specifically the intricate interactions between cellular infiltration, immune reactions, coagulation cascades, and autoantibodies (13, 14). Current urticaria treatments aim for complete response, utilizing second-generation H1 antihistamines, omalizumab, cyclosporine, and new methods primarily targeting mediators, signaling pathways, and receptors in mast cells and other immune cells (15).

Mendelian randomization (MR) is a robust epidemiological method that uses genetic variants as instrumental variables (IVs) to infer causal relationships between exposures and outcomes (16–19). This technique minimizes the influence of confounding factors, thereby enhancing the reliability of causal inferences. By leveraging genetic data, MR can effectively address biases commonly encountered in observational studies (20). Bidirectional MR analysis, also known as reciprocal MR, extends the conventional two-sample MR approach by interchanging the roles of the exposure and outcome variables to investigate potential reverse causality (21). This approach utilizes genetic variants that are significantly associated with the outcome variable as IVs to explore whether the outcome also affects exposure. In our study, this advanced analytical approach provided a deeper understanding of the complex interactions between urticaria and specific immune cell phenotypes.

Collectively, urticaria involves recurrent activation of mast cells, T cells, eosinophils, and other immune cells around lesioned venules, with complex regulatory systems affecting mast cell functions, potentially contributing to urticaria pathogenesis (22, 23). At present, the direct causal relationship between immune cells and urticaria is currently unclear. To address this, our study utilized a bidirectional MR analysis, employing IVs associated with immune cells and urticaria, to investigate this causal relationship. Genome-wide Association Study (GWAS) data for immune cells and urticaria were acquired from reputable. We selected significant single-nucleotide polymorphisms (SNPs) associated with various immune cell phenotypes as IVs, and pruned SNPs using the clump program. Statistical analysis was performed using R 4.4.0 software, employing five methods to assess the causal relationship. We addressed potential horizontal pleiotropy and outliers using MR-Egger and MR-PRESSO methods, respectively. The robustness of the results was visually assessed and validated through scatter plots, funnel plots, and a leave-one-out analysis. Our bidirectional MR analysis could potentially identify theoretical basis for urticaria-targeted therapy by elucidating the role of immune cells.

## 2 Materials and methods

### 2.1 Study design

**Figure 1:**
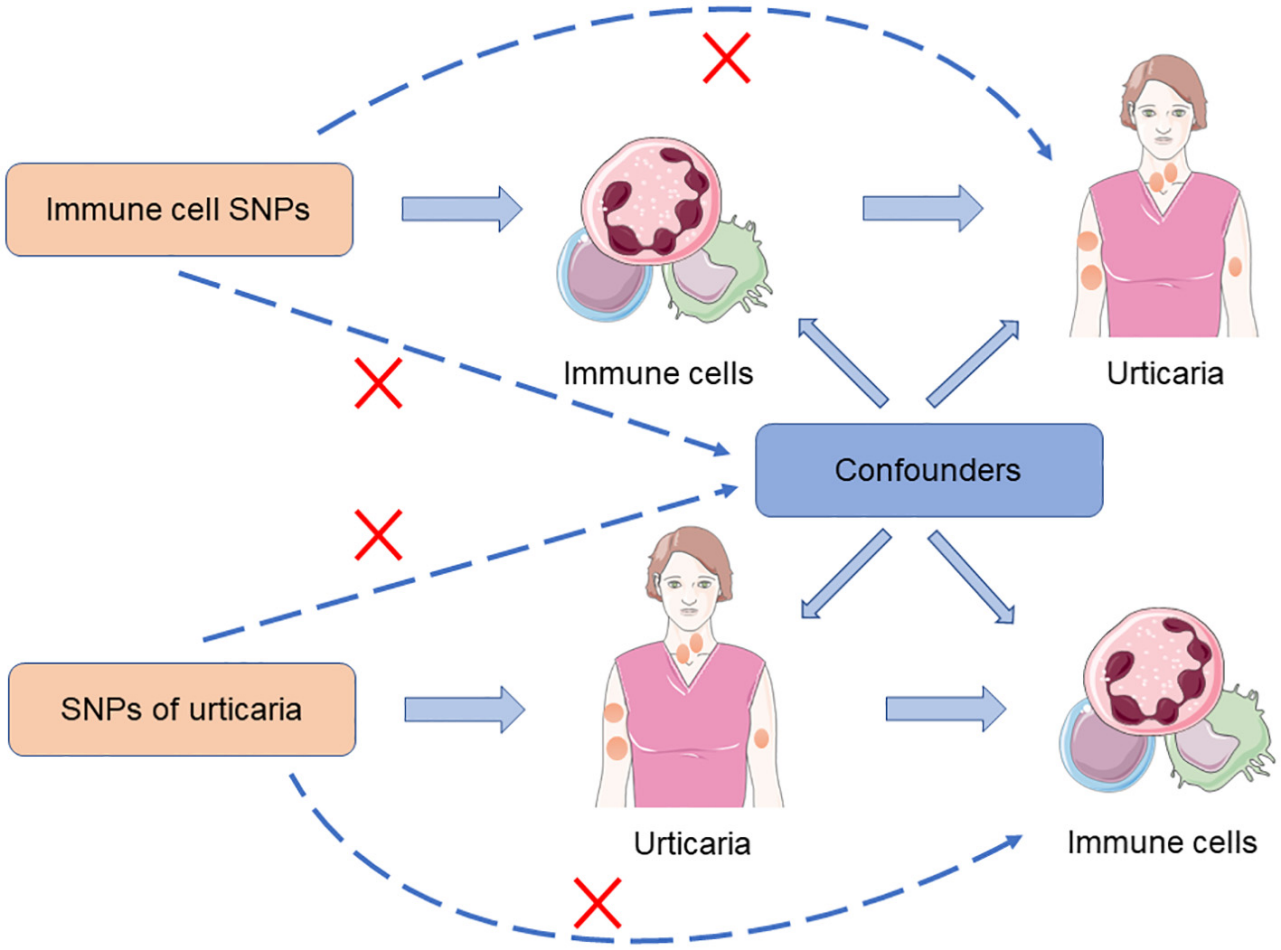
The design scheme for bidirectional MR analysis exploring the causal relationship between immune cells and urticaria.

To investigate the causal relationship between immune cells and urticaria, we performed a bidirectional MR analysis. The design scheme is shown in **Figure 1**. MR analysis relies on three key assumptions to provide valid causal inference: (1) the instrumental variable (IV) chosen, typically a genetic variant, is strongly associated with the exposure of interest; (2) the IV is not associated with any confounding factors that could bias the estimated causal effect; and (3) the IV affects the outcome solely through its impact on the exposure, without any direct or indirect pathways (24). In our study, we first deduced the causal relationship between immune cells and urticaria by selecting IVs of immune cells. Subsequently, we chose IVs associated with urticaria to deduce the causal relationship between urticaria and each selected immune cell.

### 2.2 Genome-wide Association Study data sources for immune cells

The GWAS data on immune cells were acquired from the GWAS Catalog database (www.ebi.ac.uk/gwas, accession numbers: from GCST90001391 to GCST90002121). This data included 3,757 cases and 3,027 controls with the ages ranging from 18 to 102 years. A total of 731 immunophenotypes included 32 morphological parameters (MPs), 118 absolute cell (AC) counts, 192 relative cell (RC) counts, and 389 median fluorescence intensities (MFIs) (25). This study reported 22 million genetic variations for 731 immune cell phenotypes.

### 2.3 GWAS data sources for urticaria

The GWAS data for urticaria were obtained from the FinnGen database, which can be accessed at https://www.finngen.fi/fi. This comprehensive dataset consists of information from 5,066 individuals diagnosed with urticaria and 212,464 control individuals without the condition. A total of 16,380,466 SNPs were included in the dataset. In our research, the urticaria diagnosed with urticaria included patients with allergic urticaria, idiopathic urticaria, urticaria due to cold and heat, dermatographic urticaria, vibratory urticaria, cholinergic urticaria, contact urticaria, and other and unspecified urticaria. To identify the cases of urticaria, the M13 code from the International Classification of Diseases-Tenth Revision (ICD-10) was utilized. This code is specifically associated with urticaria, ensuring that the cases included in the dataset are accurately diagnosed. All populations included in the FinnGen database are of European origin, providing a homogeneous genetic background for the analysis.

### 2.4 Instrumental variables selection

A loose cutoff value of P < 1 × 10^−5^ has been commonly employed to identify significant SNPs associated with various immune cell phenotypes. Subsequently, we employed the clump program to prune SNPs (linkage disequilibrium (LD) r^2^ < 0.01, window size > 10,000 kb). To further refine the IVs and ensure their suitability for the analysis, IVs with low F statistics (< 10) were removed. This step helps to eliminate weak instruments that may introduce bias in the MR analysis. The same IV filtering method was used for reverse MR analysis.

### 2.5 Statistical analysis

All statistical analyses were conducted using R 4.4.0 software. To assess the causal relationship between the 731 immunophenotypes and urticaria, 5 methods were employed, including MR egger, weighted median, inverse variance weighting (IVW), simple mode, and weighted mode methods. These analyses were performed using the “TwoSampleMR” package. The MR-Egger method was employed to address the potential issue of horizontal pleiotropy. This method examines the intercept term and determines if it is statistically significant, indicating the presence of horizontal pleiotropy. Additionally, the MR-PRESSO method was utilized to identify and exclude any potential outliers that could substantially influence the estimation results. MR-Egger is a method used to assess causal relationships, particularly when there may be associations between genetic instruments and confounding factors. In MR analysis, the MR-Egger method is not only used to estimate causal effects but also specifically to detect horizontal pleiotropy. The slope estimate from the MR-Egger regression reflects the causal effect, while the intercept term is used to detect horizontal pleiotropy. If the intercept term is significantly different from zero, it indicates the presence of unobserved confounding factors or horizontal pleiotropy that may affect the validity of the results. In this way, the MR-Egger method helps researchers identify and correct for potential biases in causal inference, thereby enhancing the reliability of study findings (17).MR-PRESSO is a method designed to detect and correct for pleiotropy. MR-PRESSO identifies and removes outliers to improve the precision of causal effect estimates. The method first calculates the effects of all instrumental variables and then identifies those that significantly deviate from the expected effects, treating them as outliers. By removing these outliers, MR-PRESSO provides more reliable estimates of causal effects and assesses the impact of pleiotropy on the results (26). Scatter plots and funnel plots were examined to visually assess the stability and reliability of the results. Scatter plots were used to identify any potential outliers that could impact the results. Funnel plots, on the other hand, were employed to assess the presence of heterogeneity and demonstrate the robustness of the correlation between the immunophenotypes and urticaria. The leave-one-out analysis was performed to assess the robustness of the MR findings.

## 3 Results

### 3.1 The impact of immune phenotype on the causal relationship of urticaria

**Figure 2:**
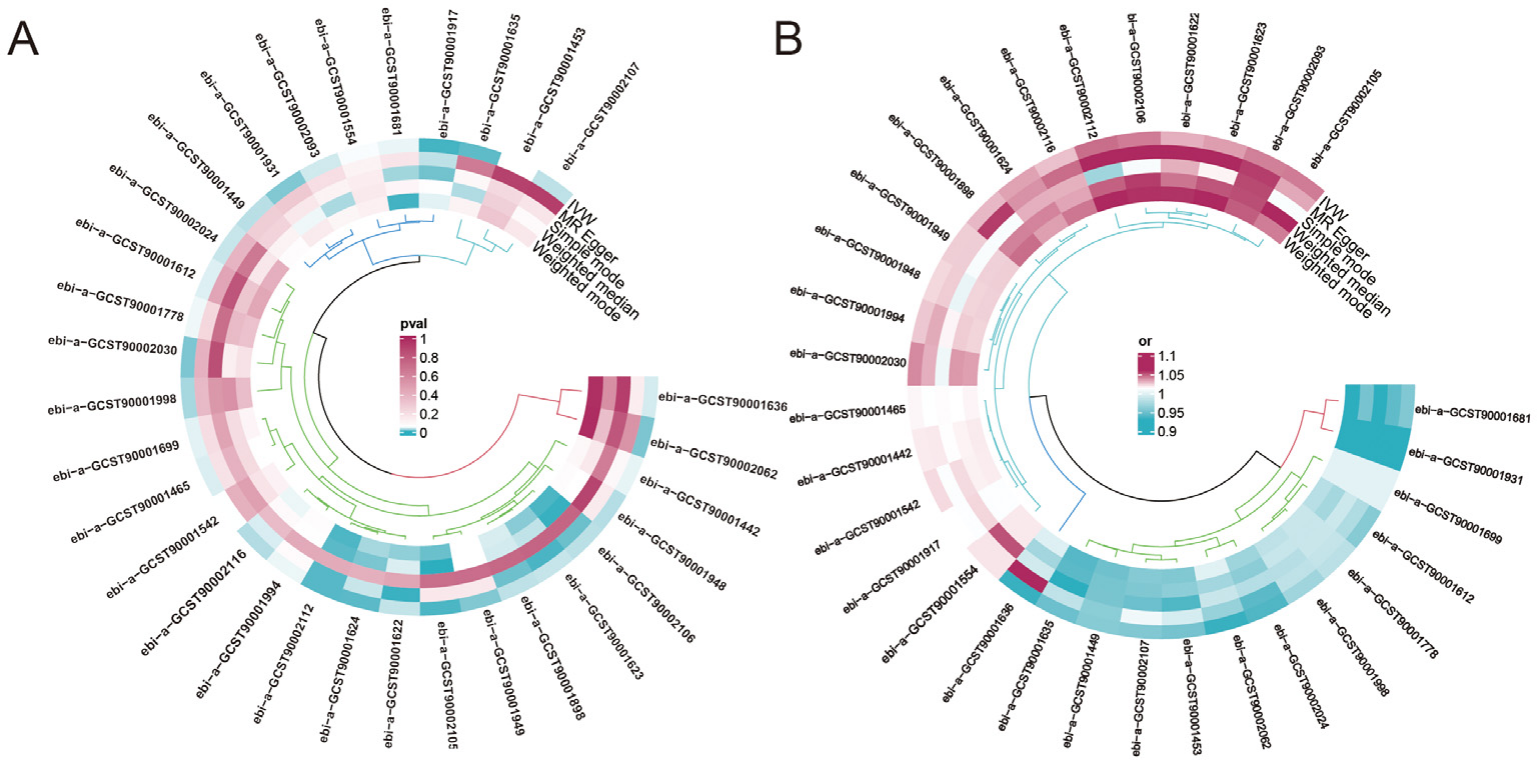
The impact of immune phenotype on the causal relationship of urticaria. **(A)** The heat map represented the ID of the positive immune cell phenotype and the P-values of the five different analysis methods including MR egger, weighted median, inverse variance weighting (IVW), simple mode, and weighted mode analysis. **(B)** The heat map represented the ID of the positive immune cell phenotype and the odds ratio(OR) of the five different analysis methods.

To investigate the causal relationship between urticaria and immune cells, the two-sample MR analysis was performed, and the IVW technique served as the primary analytical strategy. We selected immunophenotypes with p < 0.05 in IVW analysis. A total of 31 immunophenotypes are linked with urticaria (**Figure 2**; **Supplementary Figure S1**). Among them, 18 types of immunophenotypes were associated with a heightened risk of urticaria, while 13 types of immunophenotypes showed protective connections with urticaria.

**Figure 3:**
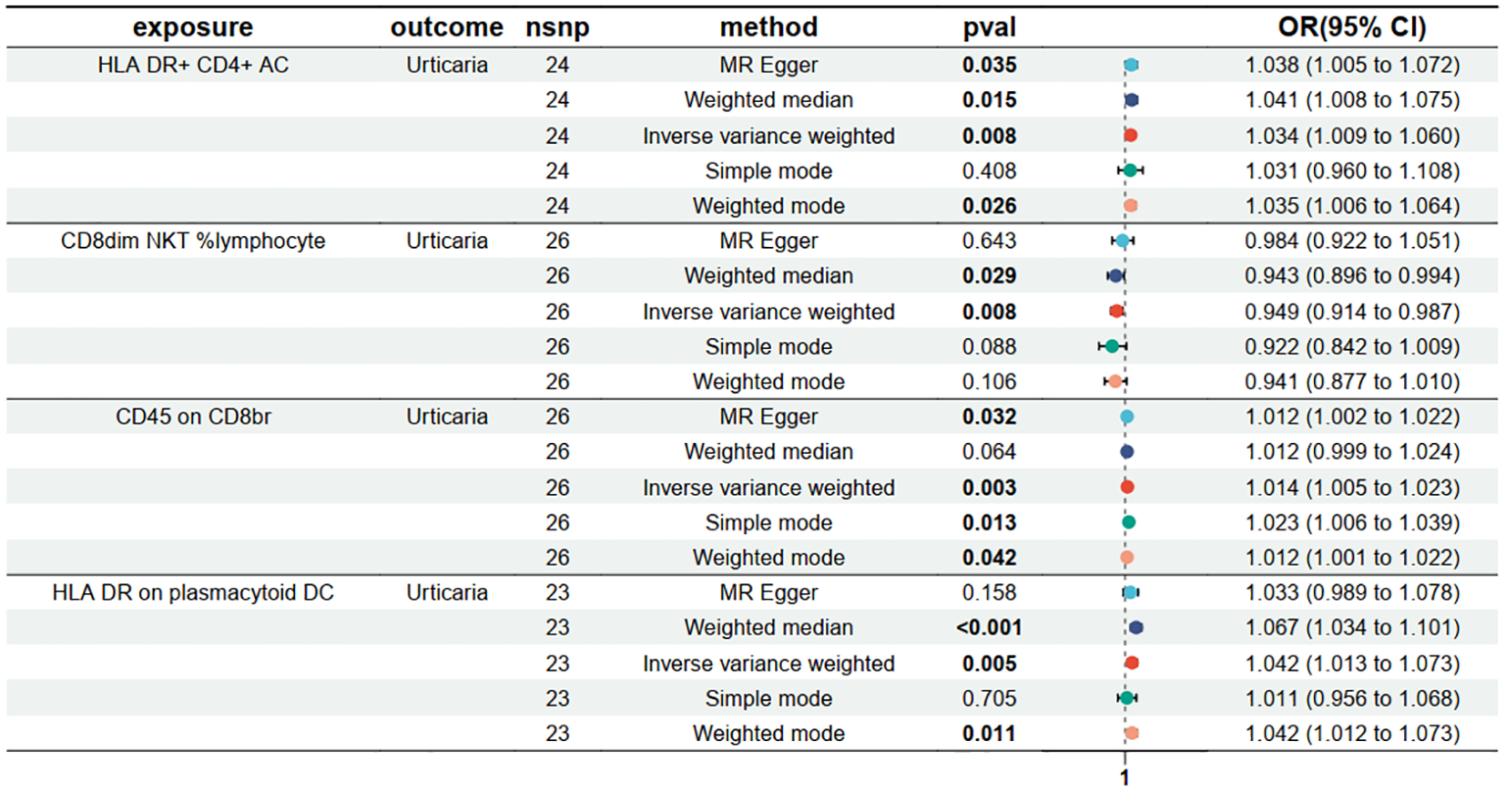
Forest plots depicting the causal effect of the 4 positive immunophenotypes on urticaria. nsnp, nonsynonymous single-nucleotide polymorphism; OR, odds ratio; CI, confidence interval.

To find immune cells with a strong causal relationship with urticaria, we set up three conditions to screen immune cells: (1) p < 0.01 in IVW analysis; (2) the same direction of OR values in five analysis methods; (3) p > 0.05 in the pleiotropy test. 4 types of immunophenotypes were found to have a causal relationship to urticaria. HLA DR+ CD4+AC [IVW: odds ratio (OR) 1.0341, 95% confidence interval (CI) 1.0089–1.0599; p = 0.0076], CD45 on CD8br [IVW: odds ratio (OR) 1.0137, 95% confidence interval (CI) 1.0046–1.0231; p = 0.0033], and HLA DR on plasmacytoid DC [IVW: odds ratio (OR) 1.0424, 95% confidence interval (CI) 1.0128–1.0729; p = 0.0047] were risk against urticaria. CD8dim NKT %lymphocyte [IVW: odds ratio (OR) 0.9495, 95% confidence interval (CI) 0.9137–0.9867; p = 0.0082] was a protective factor for urticaria (**Figure 3**).

### 3.2 Sensitivity analyses

**Figure 4:**
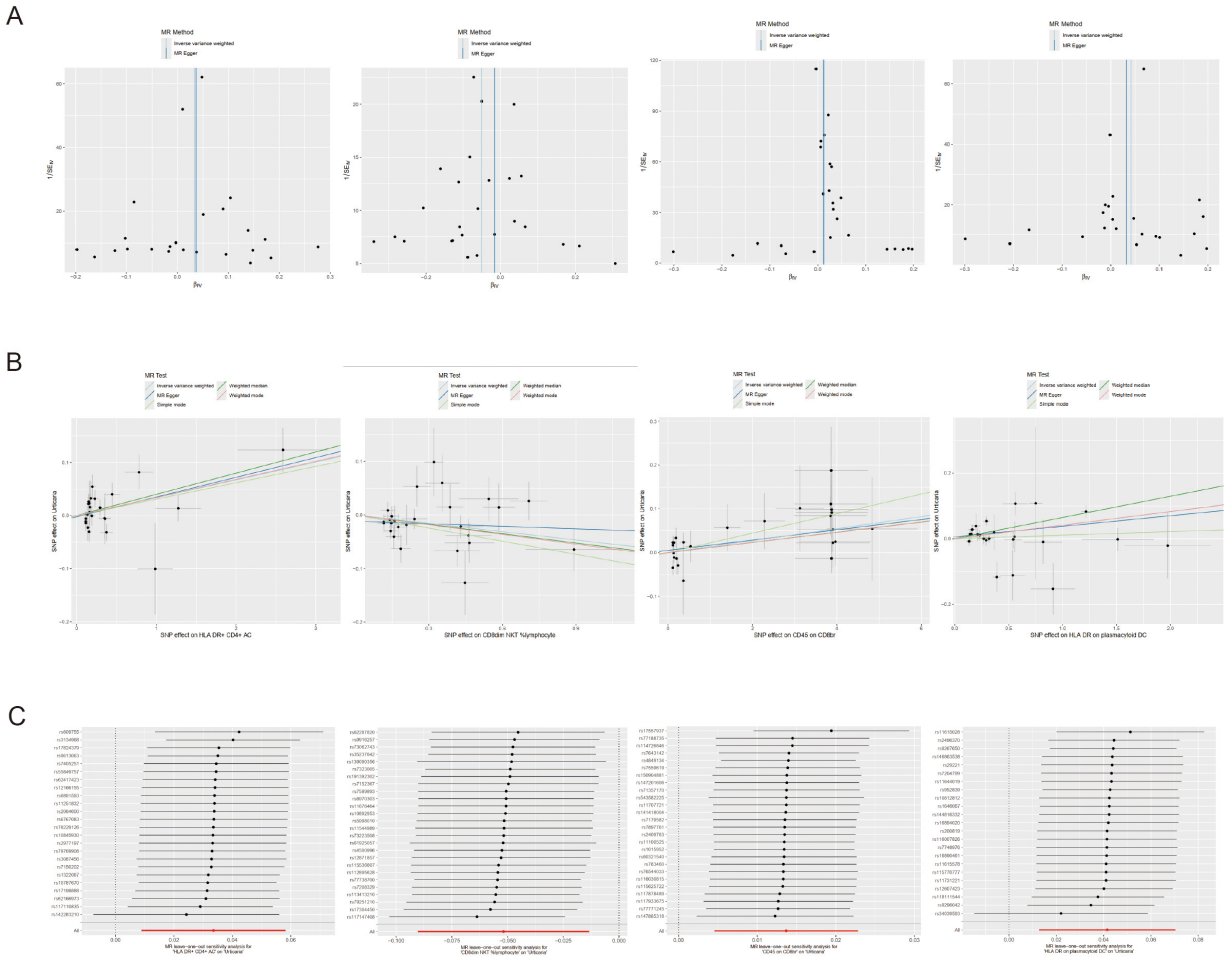
Sensitivity analyses of the causal effect of the 4 positive immunophenotypes on urticaria. **(A)** Funnel plot showing the overall heterogeneity of MR analysis of the 4 immunophenotypes on urticaria. **(B)** Scatter plots of the 4 immunophenotypes associated with urticaria. **(C)** The “leave-one-out” analysis for the causal relationship of the 4 immunophenotypes on urticaria.

The MR-Egger intercept test was conducted to assess the presence of horizontal pleiotropy in the relationships between urticaria and the four immunophenotypes. The results of this test provided no evidence of horizontal pleiotropy, indicating that the observed associations were unlikely to be influenced by confounding factors or alternative causal pathways (**Supplementary Table S1**). To further evaluate the stability of the results, scatter plots, and funnel plots were examined, which visually demonstrated the data stability (**Figures 4A, B**). Additionally, a leave-one-out analysis was performed to assess the robustness of the MR findings (**Figure 4C**). This analysis involved systematically excluding each SNP associated with the immunophenotypes and urticaria, one at a time, and evaluating the impact on the overall results. The leave-one-out analysis revealed that the exclusion of any single SNP did not significantly alter the overall findings, providing further support for the reliability and validity of the MR results.

### 3.3 The influence of psoriasis on immune cells

**Figure 5:**
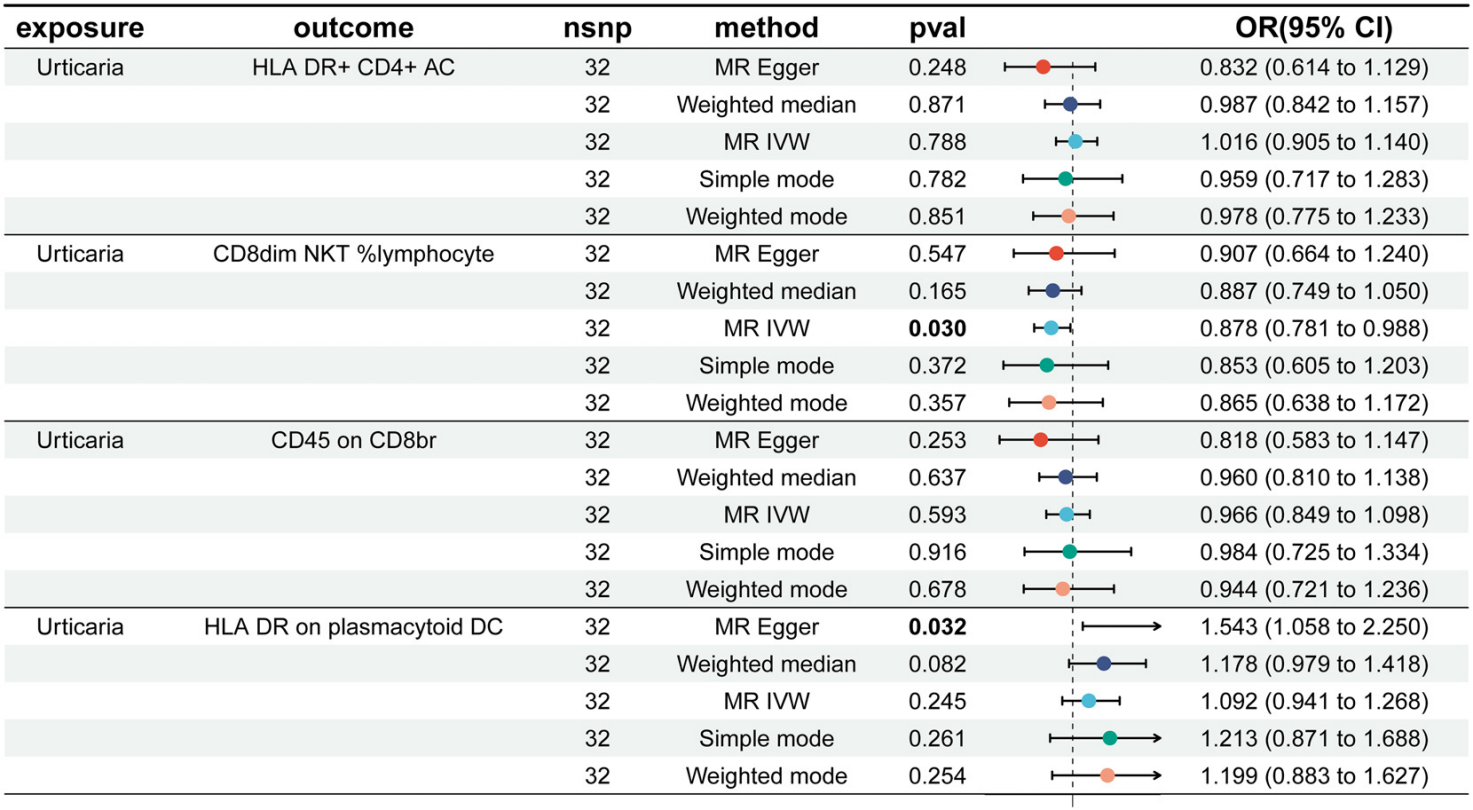
Forest plots showed the causal effect of urticaria on the 4 immunophenotypes. nsnp, nonsynonymous single-nucleotide polymorphism; OR, odds ratio; CI, confidence interval.

**Figure 6:**
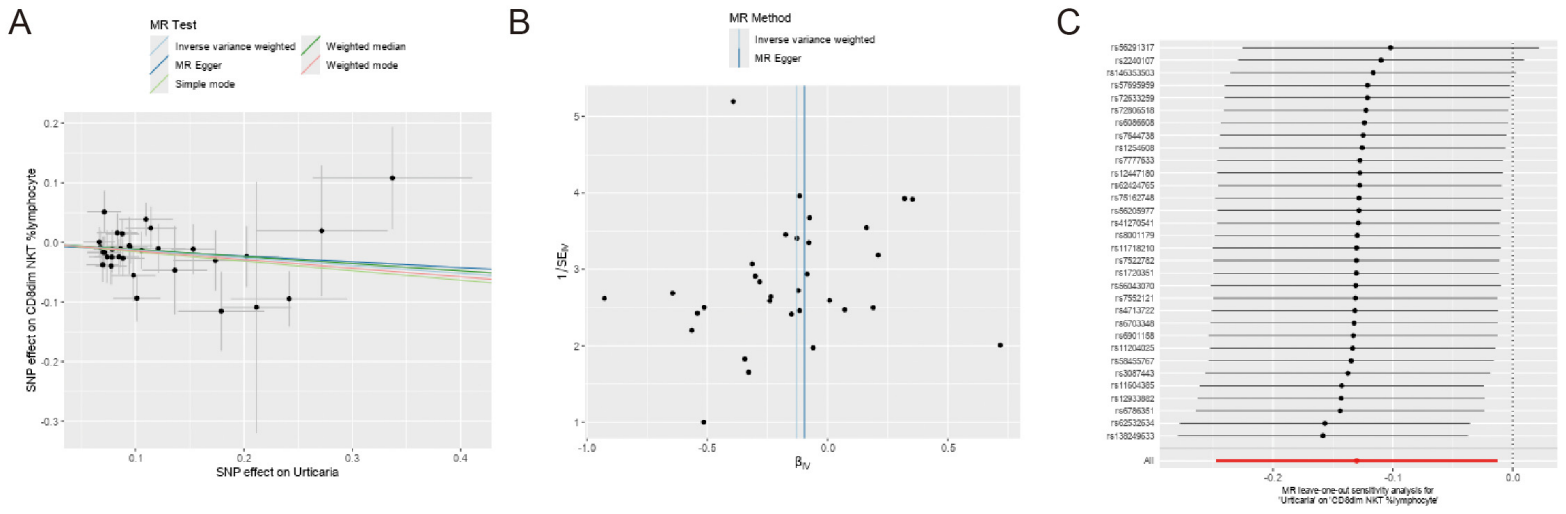
Sensitivity analyses of the causal effect of urticaria on the 4 immunophenotypes. **(A)** Funnel plot showing the overall heterogeneity of MR analysis of CD8dim NKT %lymphocyte on urticaria. **(B)** Scatter plots of genetic association between CD8dim NKT %lymphocyte and urticaria. **(C)** The “leave-one-out” analysis for the causal relationship of CD8dim NKT %lymphocyte on urticaria.

In reverse Mendelian analyses, urticaria is used as an exposure factor to explore the reverse causality between urticaria and the four immunophenotypes. According to the IVW method, urticaria had a negative causal effect on CD8dim NKT %lymphocyte [IVW: odds ratio (OR) 0.878, 95% confidence interval (CI) 0.781–0.988; p = 0.030]. But, the other 4 different analyses showed no meaningful results (**Figure 5**). The MR-Egger intercept test results showed no evidence of horizontal pleiotropy. Scatter plots and funnel plots confirmed the data stability (**Figures 6A, B**). The leave-one-out analysis further validated the robustness of the findings (**Figure 6C**).

## 4 Discussion

Urticaria is a complex skin condition characterized by the sudden appearance of itchy, red, raised bumps or welts on the skin, driven by mast cell-mediated allergic reaction (27). Our study employed a bidirectional MR analysis, using IVs associated with immune cells and urticaria, to investigate the causal relationship between immune cells and urticaria. We identified that HLA DR+ CD4+AC, CD45 on CD8br, and HLA DR on plasmacytoid DCs were associated with an increased risk of urticaria, while CD8dim NKT %lymphocyte served as a protective factor against urticaria. Furthermore, reverse MR analysis suggested an inverse causal effect of urticaria on CD8dim NKT %lymphocyte, highlighting the bidirectional nature of the relationship between urticaria and immune cell phenotypes.

The diagnosis and prevention of diseases are inseparable from the analysis of causality and markers (28). MR analysis has been a popular tool in genetic epidemiology for inferring causality between genetic predispositions and disease manifestations, including various skin diseases (29, 30). At present, there are only a few reports on MR Analysis of urticaria (31). For instance, Shi et al. confirmed a bidirectional causal relationship between gut microbiota composition and urticaria, with specific microbial genera associated with either increased risk or protective effects against the condition (32). This conclusion was supported by consistent results across multiple sensitivity analyses. In addition, Yang et al. identify urticaria as a potential risk factor for systemic lupus erythematosus (SLE), using bidirectional MR (33). They emphasized the need for increased surveillance of urticaria in patients with rheumatoid arthritis (RA) and heightened the awareness of SLE risk in individuals suffering from urticaria. And, MR analysis using data from European populations indicated a causal relationship between urticaria and an increased risk of rheumatoid arthritis, supported by robust sensitivity analyses and no detected pleiotropy (32). These studies collectively demonstrate the utility of MR in establishing causal relationships between urticaria and other conditions, highlighting consistent findings across various sensitivity analyses and emphasizing the need for disease surveillance and awareness. Given that these studies do not directly address immune mechanisms, further MR analysis is necessary to explore the specific immune-related genetic factors influencing urticaria.

Immune cells play a pivotal role in the pathogenesis of cancers, mental diseases, inflammatory diseases, and skin diseases (34–36). Subsequently, our bidirectional MR analysis revealed a significant relationship where certain immune cell phenotypes influence the risk of developing urticaria. Specifically, we identified that HLA DR+ CD4+AC, CD45 on CD8br, and HLA DR on plasmacytoid DCs were associated with an increased risk of urticaria. Conversely, CD8dim NKT %lymphocyte emerged as a protective factor, reducing the risk of this condition. These results underscore the multifaceted nature of immune involvement in urticaria, suggesting that different immune cells can either exacerbate or mitigate the disease.

Specifically, HLA DR+ CD4+AC are activated CD4+ T cells characterized by the expression of HLA-DR, and the presence of these cells may lead to an intensified immune response, resulting in excessive inflammation (37). This inflammation might trigger and exacerbate urticaria symptoms, such as wheals and itching, through the release of various pro-inflammatory factors (38). CD45 on CD8br cells indicates a state of heightened activation and proliferation (39). These cells may contribute to urticaria by directly attacking target cells or tissues and releasing inflammatory mediators, thus sustaining and intensifying the inflammatory milieu. HLA DR on plasmacytoid DCs play a crucial role in antigen presentation and immune regulation (40). The expression of HLA DR may enhance their ability to present antigens, leading to an exaggerated immune response to otherwise harmless antigens. This may activate autoreactive T cells, releasing cytokines that drive the inflammatory processes in urticaria. CD8dim NKT cells bridge innate and adaptive immunity and are known for their immunoregulatory functions (41). By secreting anti-inflammatory cytokines like IL-10, they help balance the immune system and prevent excessive inflammation. Their protective effect may reduce the frequency and severity of urticaria by suppressing hyperactive immune responses. Enhancing their function or quantity could offer a novel therapeutic strategy for restoring immune balance in urticaria.

Moreover, the reverse MR analysis suggested an inverse causal effect of urticaria on the CD8dim NKT %lymphocyte phenotype. This finding highlights the bidirectional nature of the relationship between urticaria and immune cell phenotypes, indicating that not only do certain immune cells influence the development of urticaria, but the presence of urticaria can also alter immune cell distributions or functions. This reciprocal influence enriches our understanding of urticaria pathogenesis and suggests that the disease itself may modulate immune system behavior in a way that could influence its own progression or resolution.

Despite the valuable insights gained from this study, there are several limitations and perspectives for subsequent studies. Firstly, the absence of biological validation in laboratory settings constrains our comprehension of the specific interactions between immune cells and urticaria (42). Future research should incorporate *in vitro* and *in vivo* experiments to elucidate these mechanisms and inform the development of targeted therapeutic strategies (43). Secondly, the generalizability of the findings is limited by the predominant focus on European populations. Validating these results in more diverse racial and ethnic groups is essential to ensure broader applicability and to deepen our understanding of urticaria across different demographics (44). Additionally, the bidirectional causal relationship between urticaria and immune cells remains underexplored, potentially hindering the identification of novel therapeutic targets. The stringent criteria used for selecting IVs, although ensuring robust results, might also have inadvertently excluded some specific immune phenotypes of potential relevance to urticaria. Investigating this relationship further could decipher novel treatment avenues and support the development and testing of immune cell-targeted therapies for urticaria (45).

It is worth noting that the genetic risk factors associated with urticaria have not been thoroughly examined, which may limit the effectiveness of prediction and prevention strategies. Future studies should delve into these genetic aspects to enhance personalized medical approaches for managing urticaria more effectively. Lastly, the reliance on publicly available databases may limit the type and quality of data that could be obtained, potentially missing out on important nuances that could influence the interpretation of results (46). Continuous research efforts should focus on integrating high-quality, proprietary datasets. Additionally, conducting primary data collection could provide more detailed insights and enhance the robustness of findings. The ongoing investigations by addressing these challenges, can provide a deeper understanding of the pathophysiology of urticaria for superior outcomes.

## 5 Conclusion

In conclusion, our MR analysis results indicated that HLA DR+ CD4+AC, CD45 on CD8br, and HLA DR on plasmacytoid DC increased the risk of urticaria, but CD8dim NKT %lymphocyte may lead to decreased risk of urticaria. Additionally, reverse Mendelian analysis results indicate that urticaria had an inverse causal association with CD8dim NKT %lymphocyte. These findings might aid in developing novel immune cell-targeted approaches for urticaria.

## Data Availability

The original contributions presented in the study are included in the article/**Supplementary Material**. Further inquiries can be directed to the corresponding author.

## Supplementary material

Supplementary Figure 1 Forest plots depicting the causal effect of the 31 positive immunophenotypes on urticaria.

Supplementary Table 1 Directional horizontal pleiotropy assessed by intercept term in MR Egger regression.

## References

[1] Chu CY Cho YT Jiang JH Chang CC Liao SC Tang CH. Patients with chronic urticaria have a higher risk of psychiatric disorders: a population-based study. Br J Dermatol. (2020) 182:335–41. doi: 10.1111/bjd.18240 31220338

[2] Fricke J Ávila G Keller T Weller K Lau S Maurer M. Prevalence of chronic urticaria in children and adults across the globe: Systematic review with meta-analysis. Allergy. (2020) 75:423–32. doi: 10.1111/all.14037 31494963

[3] Bernstein JA Maurer M Saini SS. BTK signaling—a crucial link in the pathophysiology of chronic spontaneous urticaria. J Allergy Clin Immunol. (2024) 153:1229–40. doi: 10.1016/j.jaci.2023.12.008 38141832

[4] Gonçalo M Gimenéz-Arnau A Al-Ahmad M Ben-Shoshan M Bernstein JA Ensina LF. The global burden of chronic urticaria for the patient and society*. Br J Dermatol. (2021) 184:226–36. doi: 10.1111/bjd.19561 32956489

[5] Yang Y Guo L Wang Z Liu P Liu X Ding J. Urticaria. Nat Rev Dis Prim. (2022) 8:62. doi: 10.1038/s41572-022-00395-1

[6] Kaplan A Lebwohl M Giménez-Arnau AM Hide M Armstrong AW Maurer M. Chronic spontaneous urticaria: Focus on pathophysiology to unlock treatment advances. Allergy. (2023) 78:389–401. doi: 10.1111/all.15603 36448493

[7] Zhang X Song X Zhang M Li C Huang Z Liu B. Prevalence and risk factors of chronic urticaria in China: A nationwide cross-sectional study. Allergy. (2022) 77:2233–6. doi: 10.1111/all.15287 35332543

[8] Zheng H Xiao X-J Shi Y-Z Zhang L-X Cao W Zheng Q-H. Efficacy of acupuncture for chronic spontaneous urticaria. Ann Intern Med. (2023) 176:1617–24. doi: 10.7326/M23-1043 37956431

[9] Gao P Rao Z Li M Sun X Gao Q Shang T. Tetrandrine represses inflammation and attenuates osteoarthritis by selective inhibition of COX-2. Curr Med Sci. (2023) 43:505–13. doi: 10.1007/s11596-023-2725-6 37204627

[10] Zhang Z Hu Y Chen Y Chen Z Zhu Y Chen M. Immunometabolism in the tumor microenvironment and its related research progress. Front Oncol. (2022) 12:1024789. doi: 10.3389/fonc.2022.1024789 36387147 PMC9659971

[11] Segú-Vergés C Gómez J Terradas-Montana P Artigas L Smeets S Ferrer M. Unveiling chronic spontaneous urticaria pathophysiology through systems biology. J Allergy Clin Immunol. (2023) 151:1005–14. doi: 10.1016/j.jaci.2022.12.809 36587849

[12] Palomares O Elewaut D Irving PM Jaumont X Tassinari P. Regulatory T cells and immunoglobulin E: A new therapeutic link for autoimmunity? Allergy. (2022) 77:3293–308. doi: 10.1111/all.15449 35852798

[13] Bhowmik R Shaharyar MA Sarkar A Mandal A Anand K Shabana H. Immunopathogenesis of urticaria: a clinical perspective on histamine and cytokine involvement. Inflammation Res. (2024) 73:877–96. doi: 10.1007/s00011-024-01869-6 38555555

[14] Worm M Vieths S Mahler V. An update on anaphylaxis and urticaria. J Allergy Clin Immunol. (2022) 150:1265–78. doi: 10.1016/j.jaci.2022.10.014 36481047

[15] Kolkhir P Giménez-Arnau AM Kulthanan K Peter J Metz M Maurer M. Urticaria. Nat Rev Dis Prim. (2022) 8:61. doi: 10.1038/s41572-022-00389-z 36109590

[16] Li Z Chen Y Ke H. Investigating the causal relationship between gut microbiota and crohn’s disease: A mendelian randomization study. Gastroenterology. (2024) 166:354–5. doi: 10.1053/j.gastro.2023.08.047 37678500

[17] Burgess S Thompson SG. Interpreting findings from Mendelian randomization using the MR-Egger method. Eur J Epidemiol. (2017) 32:377–89. doi: 10.1007/s10654-017-0255-x PMC5506233 28527048

[18] Li J Cheng C Zhang J. Autoimmune diseases and the risk of bladder cancer: A Mendelian randomization analysis. J Autoimmun. (2024) 146:103231. doi: 10.1016/j.jaut.2024.103231 38692170

[19] Levin MG Burgess S. Mendelian randomization as a tool for cardiovascular research. JAMA Cardiol. (2024) 9:79. doi: 10.1001/jamacardio.2023.4115 37938820

[20] Natarajan P Yancy CW. Mendelian randomization and cardiovascular disease—Enabling expert readership. JAMA Cardiol. (2024) 9:89. doi: 10.1001/jamacardio.2023.4126 37938849

[21] Li W Xu J-W Chai J-L Guo C-C Li G-Z Gao M. Complex causal association between genetically predicted 731 immunocyte phenotype and osteonecrosis: A bidirectional Two-sample mendelian randomization analysis. Int J Surg. (2024) 144:646–74. doi: 10.1097/JS9.0000000000001327 PMC11175804 38498404

[22] Kolkhir P Muñoz M Asero R Ferrer M Kocatürk E Metz M. Autoimmune chronic spontaneous urticaria. J Allergy Clin Immunol. (2022) 149:1819–31. doi: 10.1016/j.jaci.2022.04.010 35667749

[23] Rutter KJ Peake M Hawkshaw NJ Scholey R Bulfone-Paus S Friedmann PS. Solar urticaria involves rapid mast cell STAT3 activation and neutrophil recruitment, with FcϵRI as an upstream regulator. J Allergy Clin Immunol. (2024) 153:1369–1380.e15. doi: 10.1016/j.jaci.2023.12.021 38184075

[24] Bowden J Holmes MV. Meta-analysis and Mendelian randomization: A review. Res Synth Methods. (2019) 10:486–96. doi: 10.1002/jrsm.1346 PMC6973275 30861319

[25] Orrù V Steri M Sidore C Marongiu M Serra V Olla S. Complex genetic signatures in immune cells underlie autoimmunity and inform therapy. Nat Genet. (2020) 52:1036–45. doi: 10.1038/s41588-020-0684-4 PMC8517961 32929287

[26] Verbanck M Chen C-Y Neale B Do R. Detection of widespread horizontal pleiotropy in causal relationships inferred from Mendelian randomization between complex traits and diseases. Nat Genet. (2018) 50:693–8. doi: 10.1038/s41588-018-0099-7 PMC6083837 29686387

[27] Zuberbier T Aberer W Asero R Abdul Latiff AH Baker D Ballmer-Weber B. The EAACI/GA^2^LEN/EDF/WAO guideline for the definition, classification, diagnosis and management of urticaria. Allergy. (2018) 73:1393–414. doi: 10.1111/all.13397 29336054

[28] Wang Y-R Yang K Wen Y Wang P Hu Y Lai Y. Screening and diagnosis of cardiovascular disease using artificial intelligence-enabled cardiac magnetic resonance imaging. Nat Med. (2024) 30:1471–80. doi: 10.1038/s41591-024-02971-2 PMC11108784 38740996

[29] Yu N Qi H Guo Y Wu L Su J Huang K. Associations between rheumatoid arthritis and skin cancer: A bidirectional two-sample Mendelian randomization study. J Am Acad Dermatol. (2024) 90:198–200. doi: 10.1016/j.jaad.2023.09.046 37758025

[30] Ahn K Penn RB Rattan S Panettieri RA Voight BF An SS. Mendelian randomization analysis reveals a complex genetic interplay among atopic dermatitis, asthma, and gastroesophageal reflux disease. Am J Respir Crit Care Med. (2023) 207:130–7. doi: 10.1164/rccm.202205-0951OC PMC9893317 36214830

[31] Ference BA. How to use Mendelian randomization to anticipate the results of randomized trials. Eur Heart J. (2018) 39:360–2. doi: 10.1093/eurheartj/ehx462 29020392

[32] Shi Y-Z Tao Q-F Qin H-Y Li Y Zheng H. Causal relationship between gut microbiota and urticaria: a bidirectional two-sample mendelian randomization study. Front Microbiol. (2023) 14:1189484. doi: 10.3389/fmicb.2023.1189484 37426010 PMC10324650

[33] Yang M Su Y Xu K Wen P Zhang B Guo J. Common autoimmune diseases and urticaria: the causal relationship from a bidirectional two-sample mendelian randomization study. Front Immunol. (2023) 14:1280135. doi: 10.3389/fimmu.2023.1280135 38022623 PMC10652397

[34] Kabashima K Honda T Ginhoux F Egawa G. The immunological anatomy of the skin. Nat Rev Immunol. (2019) 19:19–30. doi: 10.1038/s41577-018-0084-5 30429578

[35] Yao C Zurawski SM Jarrett ES Chicoine B Crabtree J Peterson EJ. Skin dendritic cells induce follicular helper T cells and protective humoral immune responses. J Allergy Clin Immunol. (2015) 136:1387–1397.e7. doi: 10.1016/j.jaci.2015.04.001 25962902 PMC4639468

[36] Zhao Y Yang Z Fang C Xiao D Shi Y Lin Y. A single-center observational study on the expression of circulating interleukin-20 levels and predicting outcomes in human chronic heart failure: A 2-year follow-up cohort study. Clin Chim Acta. (2020) 510:5–10. doi: 10.1016/j.cca.2020.06.048 32622964

[37] Maggi J Carrascal M Soto L Neira O Cuéllar MC Aravena O. Isolation of HLA-DR-naturally presented peptides identifies T-cell epitopes for rheumatoid arthritis. Ann Rheum Dis. (2022) 81:1096–105. doi: 10.1136/annrheumdis-2021-220371 35459695

[38] Giménez-Arnau AM DeMontojoye L Asero R Cugno M Kulthanan K Yanase Y. The pathogenesis of chronic spontaneous urticaria: the role of infiltrating cells. J Allergy Clin Immunol Pract. (2021) 9:2195–208. doi: 10.1016/j.jaip.2021.03.033 33823316

[39] Lv Y. The effects of immunomodulatory drugs on cerebral small vessel disease: A mediation Mendelian randomization analysis. Int Immunopharmacol. (2024) 140:112786. doi: 10.1016/j.intimp.2024.112786 39121606

[40] Stankovic B Bjørhovde HAK Skarshaug R Aamodt H Frafjord A Müller E. Immune cell composition in human non-small cell lung cancer. Front Immunol. (2018) 9:3101. doi: 10.3389/fimmu.2018.03101 30774636 PMC6367276

[41] Wang X Gao H Zeng Y Chen J. A Mendelian analysis of the relationships between immune cells and breast cancer. Front Oncol. (2024) 14:1341292. doi: 10.3389/fonc.2024.1341292 38327747 PMC10847340

[42] Schmidt AF Finan C Gordillo-Marañón M Asselbergs FW Freitag DF Patel RS. Genetic drug target validation using Mendelian randomisation. Nat Commun. (2020) 11:3255. doi: 10.1038/s41467-020-16969-0 32591531 PMC7320010

[43] Yang C Fagan AM Perrin RJ Rhinn H Harari O Cruchaga C. Mendelian randomization and genetic colocalization infer the effects of the multi-tissue proteome on 211 complex disease-related phenotypes. Genome Med. (2022) 14:140. doi: 10.1186/s13073-022-01140-9 36510323 PMC9746220

[44] Howe LJ Lee MK Sharp GC Davey Smith G St Pourcain B Shaffer JR. Investigating the shared genetics of non-syndromic cleft lip/palate and facial morphology. PloS Genet. (2018) 14:e1007501. doi: 10.1371/journal.pgen.1007501 30067744 PMC6089455

[45] Chen B Yan Y Wang H Xu J. Association between genetically determined telomere length and health-related outcomes: A systematic review and meta-analysis of Mendelian randomization studies. Aging Cell. (2023) 22:34–48. doi: 10.1111/acel.13874 PMC10352568 37232505

[46] Aguillon-Rodriguez V Angelaki D Bayer H Bonacchi N Carandini M Cazettes F. Standardized and reproducible measurement of decision-making in mice. Elife. (2021) 10:367–7. doi: 10.7554/eLife.63711 PMC8137147 34011433

